# Prevalence of Limited Health Literacy in the Philippines: First National Survey

**DOI:** 10.3928/24748307-20220419-01

**Published:** 2022-04

**Authors:** Ma. Carmen C. Tolabing, Kim Carmela D. Co, Ophelia M. Mendoza, Nona Rachel C. Mira, Romeo R. Quizon, Ma. Sandra B. Tempongko, Martin Aaron M. Mamangon, Isabel Teresa O. Salido, Peter W.S. Chang

## Abstract

**Background:**

Health literacy (HL) is the ability to access, understand, appraise, and apply health information across the three domains of the health continuum: health care, disease prevention, and health promotion. It is needed for people to effectively manage their health. Information on population HL level is useful for crafting appropriate and targeted interventions to improve HL.

**Objective:**

The aim of this study was to describe the HL level of Filipino people at the national and subnational levels.

**Methods:**

A cross-sectional survey was conducted between 2018 and 2019 with 2,303 randomly selected Filipino people age 15 to 70 years, using an adapted Asia version of the European Health Literacy Survey Questionnaire-47. Prevalence estimates for limited HL and the corresponding 95% confidence interval (CI) were computed at the national and subnational levels.

**Key Results:**

The nationwide prevalence of limited HL was 51.5% (95% CI, [49.5%, 53.6%]), while sub-national prevalence estimates ranged from 48.2% to 65.4%. The prevalence varied across HL dimensions, with difficulty in access to information having the highest level. Similarly, prevalence across domains was variable; health care-related HL had the highest prevalence of limited HL. The HL levels for different dimensions and domains also varied across subnational groups.

**Conclusion:**

Many Filipino people had limited HL, and prevalence estimates varied across HL dimensions, HL domains, subnational groupings, and sociodemographic characteristics. The results highlight the need for targeted interventions focusing on subgroups with limited HL and on dimensions and domains where Filipino people have limited HL. **[*HLRP: Health Literacy Research and Practice*. 2022;6(2):e104–e112.]**

**Plain Language Summary:**

The National Health Literacy Survey is the first nationwide survey on the prevalence of HL in the Philippines, involving 2,303 randomly selected Filipino residents age 15 to 70 years. Many Filipino people have limited HL, and the prevalence of HL varies across the components of HL, subnational groupings, and sociodemographic characteristics, highlighting the need for targeted interventions.

Health literacy (HL) refers to the ability to access, understand, appraise, and apply health information when making judgments and decisions concerning health care, disease prevention, and health promotion (Sørensen et al., 2012). HL has been identified as a determinant of reduced morbidity, mortality, disability, and equity in health (Nutbeam, 2017). The United Nations Economic and Social Council (2009) has called for the “development of appropriate action plans to promote health literacy” (p. 6). The World Health Organization (WHO) has similarly called for action to address HL. In 2015, WHO published the Health Literacy Toolkit, which provides guidance on empowering communities and strengthening health systems (Dodson et al., 2015).

There is no existing national HL policy or program in the Philippines as of writing (Department of Health, n.d.; Senate of the Philippines, 2013). This gap may be due to lack of data on population HL, which may be provided by a national HL survey. Measuring population HL can inform the drafting of these policies and programs and facilitate the crafting of appropriate interventions, such as policy, modification of health education programs, and training of health providers to become more aware of the concept of HL (Rondia et al., 2019). Thus, the aim of this study was to describe the HL level of Filipino people age 15 to 70 years at the national and sub-national levels.

## Methods

### Study Design and Sampling

A cross-sectional study design was employed. The study population consisted of Filipino residents age 15 to 70 years. Older adults with cognitive impairment, such as problems with memory, language, and thinking, based on the Mini-Cog test for people age 60 years and older (Mini-Cog, n.d.) and those unable to consent were excluded. Multi-stage sampling was used for respondent selection. The stratification variable was the subnational grouping: Luzon, Visayas, or Mindanao. The National Capital Region (NCR), which is part of Luzon, was peculiar in that it is 100% urban with easy access to resources; thus, NCR was segregated from Luzon and was made the fourth category for the stratification variable. Within each subnational grouping, sample provinces, cities/municipalities, barangays, and households were selected by systematic sampling with probabilities proportional to size. Only one individual, age 15 to 70 years, was selected in each sample household to minimize the effect of intra-cluster homogeneity.

A total of 2,303 respondents participated in the survey. This was the minimum required sample to achieve a 95% confidence level, 50% anticipated value of the various proportions to be estimated from the survey, and margin of error with values varying from ±2% to ±7% for the national and subnational estimates. The sample size was adjusted to account for 1.5% design effect and 10% non-response. If the respondent was unavailable during the first visit, a callback was made. Of the 276 respondents requiring callbacks, 29% (*n* = 81) were subsequently replaced after three failed attempts to interview them. This represented 3.5% of the total sample size.

### Measurement

The adapted Asia version of the 47-item European Health Literacy Survey Questionnaire measured the components of HL, including its dimensions (ability to access, understand, appraise, and apply health information) and domains (health care, disease prevention, health promotion). Selected sociodemographic characteristics were also collected.

The questionnaire has been concluded to be valid and reliable in a study across six Asian countries (Duong et al., 2017). The HL classifications have been reported to be associated with known determinants (older age and lower educational attainment) and health-related outcomes of HL (not having health insurance and not visiting a doctor in the past 12 months) in the Philippine setting (Agosto et al., 2018).

The questionnaire included 47 items, each answered using a 4-point Likert-type scale. The index scores (index = (mean−1) × (50/3)) ([Duong et al., 2017]) were computed for the overall health literacy (47 items) and the dimension-specific and domain-specific health literacy (11–16 items each) (Sørensen et al., 2012). Based on the index score, a respondent was classified into one of three HL categories: *limited* (0–33), *sufficient* (>33–42), or *excellent* (>42–50) (**Table A**). These cut-offs were set by the developers according to correlation patterns between HL levels and identified covariates (Sørensen et al., 2015). They indicate gradations in ability to carry out health-related tasks (accessing, understanding, appraising, and applying) successfully as determined by an expert panel, with the *limited *category indicating more difficulties in performing these tasks. Several national surveys have used these cut-offs (Espanha & Ávila, 2016; Nakayama et al., 2015; Palumbo et al., 2016; Schaeffer et al., 2017; Sørensen et al., 2012). These cut-offs and classifications have been used to make cross-country comparisons of HL distributions (Duong et al., 2015) and have also been used in a local study among adults (Agosto et al., 2018).

**Table A:**
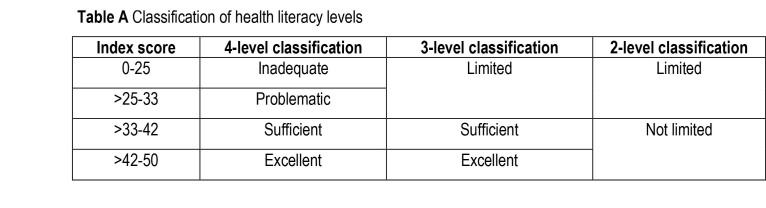
Classification of health literacy levels

**Index score**	**4-level classification**	**3-level classification**	**2-level classification**
0–25	Inadequate	Limited	Limited
>25–33	Problematic		
>33–42	Sufficient	Sufficient	Not limited
>42–50	Excellent	Excellent	

The survey was administered in multiple languages. The questionnaire underwent localization, consisting of translation, back-translation, translation analysis, and cultural adaptation corresponding to the nine major Philippine languages (AHLA Philippines, 2019). The translation analysis involved an iterative process (Hall et al., 2018) to ensure that the original concepts were preserved in translation; the cultural adaptation was carried out through focus group discussions. The localized versions were pre-tested among 59 respondents.

### Data Collection

Trained interviewers conducted face-to-face interviews using the Computer Assisted Personal Interviewing (CAPI) method from 2018 to 2019. Interviewers were locals who spoke the local language. Informed consent was obtained from each respondent.

The study was granted ethics clearance by the National Ethics Committee (NEC Code:2018-013 Tolabing-Literacy).

### Data Analysis

STATA 12 was used for data processing and analysis. Proportions and their corresponding 95% confidence intervals were computed.

## Results

### Respondent Profile

The mean age of the respondents was 40.6 ± 14.7 years, and the majority were women (73.8%), urban residents (69.9%), married (54.6%), Catholic (79.2%), and not gainfully employed (52%). About 42% attained high school, and 30.4% reported an annual income of $2,063 to $5,157 **(Table B)**.

**Table B:**
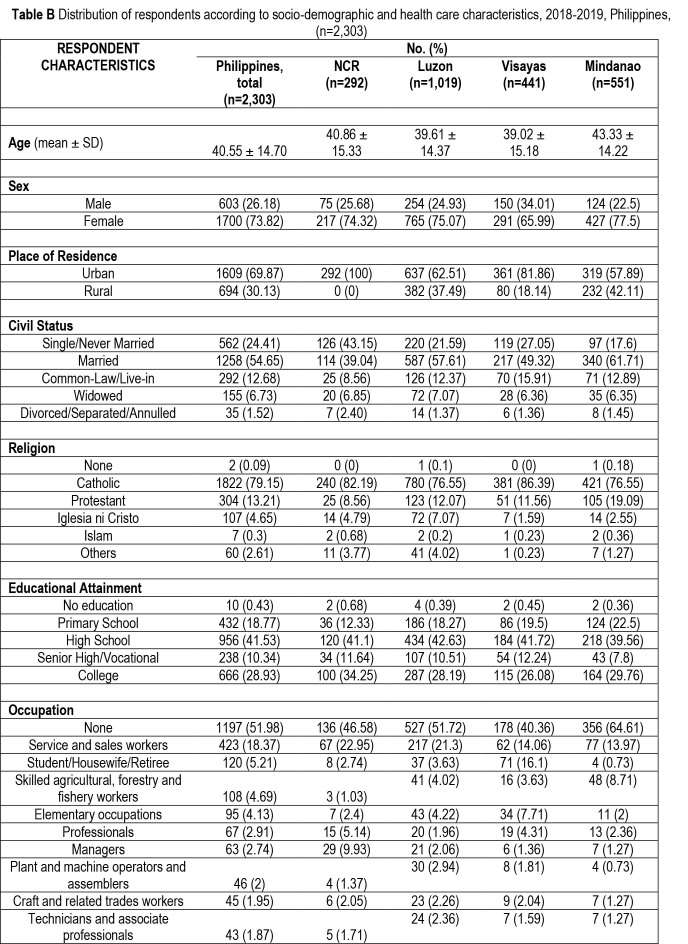

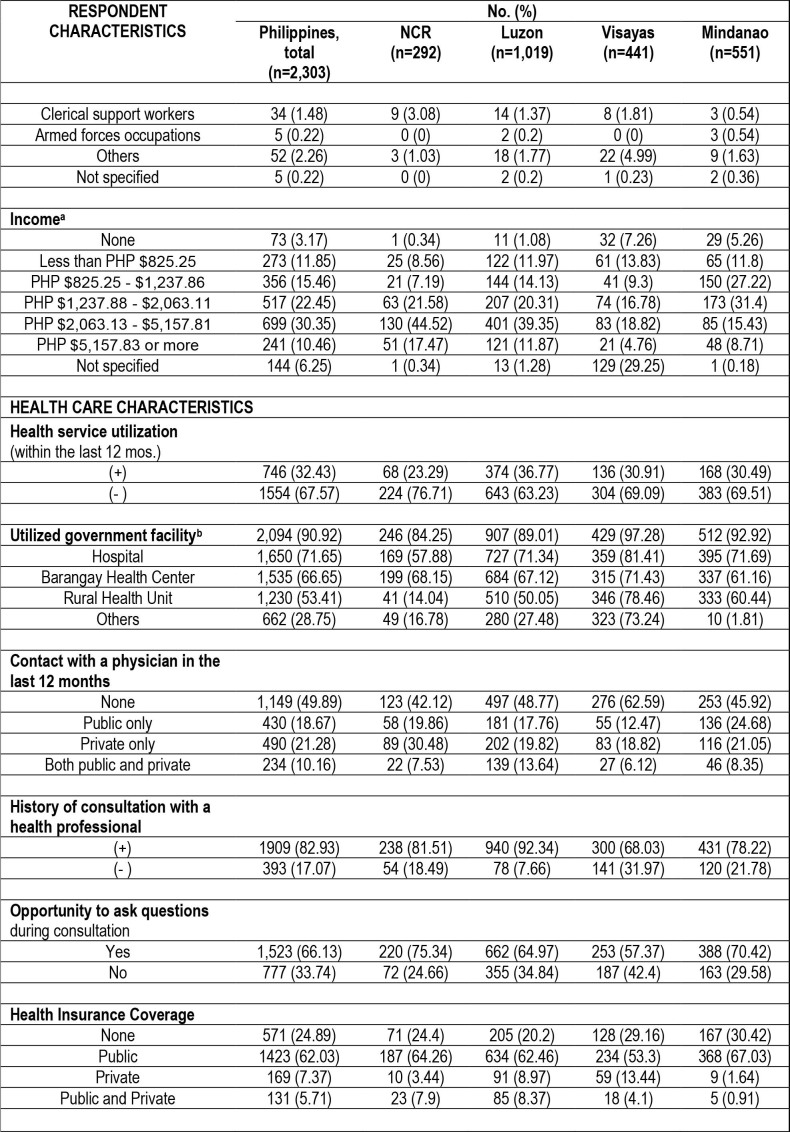

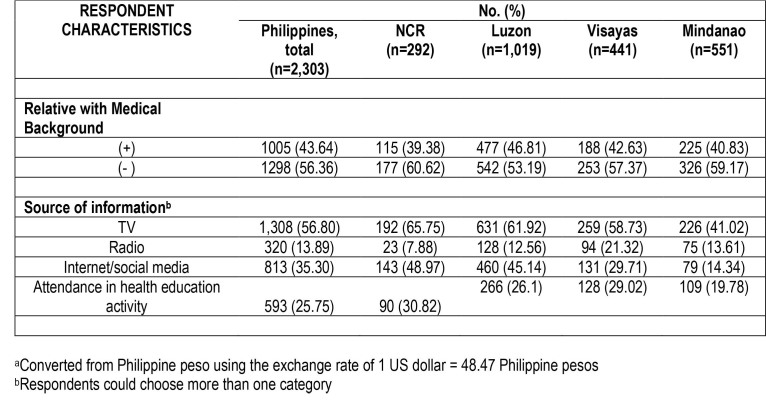
Distribution of respondents according to socio-demographic and health care characteristics, 2018–2019, Philippines, (n=2,303)

**RESPONDENT CHARACTERISTICS**	**No. (%)**				
	**Philippines, total (n=2,303)**	**NCR (n=292)**	**Luzon (n=1,019)**	**Visayas (n=441)**	**Mindanao (n=551)**
**Age** (mean ± SD)	40.55 ± 14.70	40.86 ± 15.33	39.61 ± 14.37	39.02 ± 15.18	43.33 ± 14.22
**Sex**					
Male	603 (26.18)	75 (25.68)	254 (24.93)	150 (34.01)	124 (22.5)
Female	1700 (73.82)	217 (74.32)	765 (75.07)	291 (65.99)	427 (77.5)
**Place of Residence**					
Urban	1609 (69.87)	292 (100)	637 (62.51)	361 (81.86)	319 (57.89)
Rural	694 (30.13)	0 (0)	382 (37.49)	80 (18.14)	232 (42.11)
**Civil Status**					
Single/Never Married	562 (24.41)	126 (43.15)	220 (21.59)	119 (27.05)	97 (17.6)
Married	1258 (54.65)	114 (39.04)	587 (57.61)	217 (49.32)	340 (61.71)
Common-Law/Live-in	292 (12.68)	25 (8.56)	126 (12.37)	70 (15.91)	71 (12.89)
Widowed	155 (6.73)	20 (6.85)	72 (7.07)	28 (6.36)	35 (6.35)
Divorced/Separated/Annulled	35 (1.52)	7 (2.40)	14 (1.37)	6 (1.36)	8 (1.45)
**Religion**					
None	2 (0.09)	0 (0)	1 (0.1)	0 (0)	1 (0.18)
Catholic	1822 (79.15)	240 (82.19)	780 (76.55)	381 (86.39)	421 (76.55)
Protestant	304 (13.21)	25 (8.56)	123 (12.07)	51 (11.56)	105 (19.09)
Iglesia ni Cristo	107 (4.65)	14 (4.79)	72 (7.07)	7 (1.59)	14 (2.55)
Islam	7 (0.3)	2 (0.68)	2 (0.2)	1 (0.23)	2 (0.36)
Others	60 (2.61)	11 (3.77)	41 (4.02)	1 (0.23)	7 (1.27)
**Educational Attainment**					
No education	10 (0.43)	2 (0.68)	4 (0.39)	2 (0.45)	2 (0.36)
Primary School	432 (18.77)	36 (12.33)	186 (18.27)	86 (19.5)	124 (22.5)
High School	956 (41.53)	120 (41.1)	434 (42.63)	184 (41.72)	218 (39.56)
Senior High/Vocational	238 (10.34)	34 (11.64)	107 (10.51)	54 (12.24)	43 (7.8)
College	666 (28.93)	100 (34.25)	287 (28.19)	115 (26.08)	164 (29.76)
**Occupation**					
None	1197 (51.98)	136 (46.58)	527 (51.72)	178 (40.36)	356 (64.61)
Service and sales workers	423 (18.37)	67 (22.95)	217 (21.3)	62 (14.06)	77 (13.97)
Student/Housewife/Retiree	120 (5.21)	8 (2.74)	37 (3.63)	71 (16.1)	4 (0.73)
Skilled agricultural, forestry and fishery workers	108 (4.69)	3 (1.03)	41 (4.02)	16 (3.63)	48 (8.71)
Elementary occupations	95 (4.13)	7 (2.4)	43 (4.22)	34 (7.71)	11 (2)
Professionals	67 (2.91)	15 (5.14)	20 (1.96)	19 (4.31)	13 (2.36)
Managers	63 (2.74)	29 (9.93)	21 (2.06)	6 (1.36)	7 (1.27)
Plant and machine operators and assemblers	46 (2)	4 (1.37)	30 (2.94)	8 (1.81)	4 (0.73)
Craft and related trades workers	45 (1.95)	6 (2.05)	23 (2.26)	9 (2.04)	7 (1.27)
Technicians and associate professionals	43 (1.87)	5 (1.71)	24 (2.36)	7 (1.59)	7 (1.27)
Clerical support workers	34 (1.48)	9 (3.08)	14 (1.37)	8 (1.81)	3 (0.54)
Armed forces occupations	5 (0.22)	0 (0)	2 (0.2)	0 (0)	3 (0.54)
Others	52 (2.26)	3 (1.03)	18 (1.77)	22 (4.99)	9 (1.63)
Not specified	5 (0.22)	0 (0)	2 (0.2)	1 (0.23)	2 (0.36)
**Income^a^**					
None	73 (3.17)	1 (0.34)	11 (1.08)	32 (7.26)	29 (5.26)
Less than PHP $825.25	273 (11.85)	25 (8.56)	122 (11.97)	61 (13.83)	65 (11.8)
PHP $825.25 – $1,237.86	356 (15.46)	21 (7.19)	144 (14.13)	41 (9.3)	150 (27.22)
PHP $1,237.88 – $2,063.11	517 (22.45)	63 (21.58)	207 (20.31)	74 (16.78)	173 (31.4)
PHP $2,063.13 – $5,157.81	699 (30.35)	130 (44.52)	401 (39.35)	83 (18.82)	85 (15.43)
PHP $5,157.83 or more	241 (10.46)	51 (17.47)	121 (11.87)	21 (4.76)	48 (8.71)
Not specified	144 (6.25)	1 (0.34)	13 (1.28)	129 (29.25)	1 (0.18)
**HEALTH CARE CHARACTERISTICS**					
**Health service utilization** (within the last 12 mos.)					
(+)	746 (32.43)	68 (23.29)	374 (36.77)	136 (30.91)	168 (30.49)
(−)	1554 (67.57)	224 (76.71)	643 (63.23)	304 (69.09)	383 (69.51)
**Utilized government facility^b^**	2,094 (90.92)	246 (84.25)	907 (89.01)	429 (97.28)	512 (92.92)
Hospital	1,650 (71.65)	169 (57.88)	727 (71.34)	359 (81.41)	395 (71.69)
Barangay Health Center	1,535 (66.65)	199 (68.15)	684 (67.12)	315 (71.43)	337 (61.16)
Rural Health Unit	1,230 (53.41)	41 (14.04)	510 (50.05)	346 (78.46)	333 (60.44)
Others	662 (28.75)	49 (16.78)	280 (27.48)	323 (73.24)	10 (1.81)
**Contact with a physician in the last 12 months**					
None	1,149 (49.89)	123 (42.12)	497 (48.77)	276 (62.59)	253 (45.92)
Public only	430 (18.67)	58 (19.86)	181 (17.76)	55 (12.47)	136 (24.68)
Private only	490 (21.28)	89 (30.48)	202 (19.82)	83 (18.82)	116 (21.05)
Both public and private	234 (10.16)	22 (7.53)	139 (13.64)	27 (6.12)	46 (8.35)
**History of consultation with a health professional**					
(+)	1909 (82.93)	238 (81.51)	940 (92.34)	300 (68.03)	431 (78.22)
(−)	393 (17.07)	54 (18.49)	78 (7.66)	141 (31.97)	120 (21.78)
**Opportunity to ask questions** during consultation					
Yes	1,523 (66.13)	220 (75.34)	662 (64.97)	253 (57.37)	388 (70.42)
No	777 (33.74)	72 (24.66)	355 (34.84)	187 (42.4)	163 (29.58)
**Health Insurance Coverage**					
None	571 (24.89)	71 (24.4)	205 (20.2)	128 (29.16)	167 (30.42)
Public	1423 (62.03)	187 (64.26)	634 (62.46)	234 (53.3)	368 (67.03)
Private	169 (7.37)	10 (3.44)	91 (8.97)	59 (13.44)	9 (1.64)
Public and Private	131 (5.71)	23 (7.9)	85 (8.37)	18 (4.1)	5 (0.91)
**Relative with Medical Background**					
(+)	1005 (43.64)	115 (39.38)	477 (46.81)	188 (42.63)	225 (40.83)
(−)	1298 (56.36)	177 (60.62)	542 (53.19)	253 (57.37)	326 (59.17)
**Source of information^b^**					
TV	1,308 (56.80)	192 (65.75)	631 (61.92)	259 (58.73)	226 (41.02)
Radio	320 (13.89)	23 (7.88)	128 (12.56)	94 (21.32)	75 (13.61)
Internet/social media	813 (35.30)	143 (48.97)	460 (45.14)	131 (29.71)	79 (14.34)
Attendance in health education activity	593 (25.75)	90 (30.82)	266 (26.1)	128 (29.02)	109 (19.78)

a Converted from Philippine peso using the exchange rate of 1 US dollar = 48.47 Philippine pesos

b Respondents could choose more than one category

### Health Literacy

The nationwide prevalence of limited HL was 51.5% (95% confidence interval [CI], [49.5%, 53.6%]). NCR and Luzon had the highest (65.4%) and the lowest (48.2%) prevalence, respectively (**Table 1** and **Table C)**.

**Table 1:**
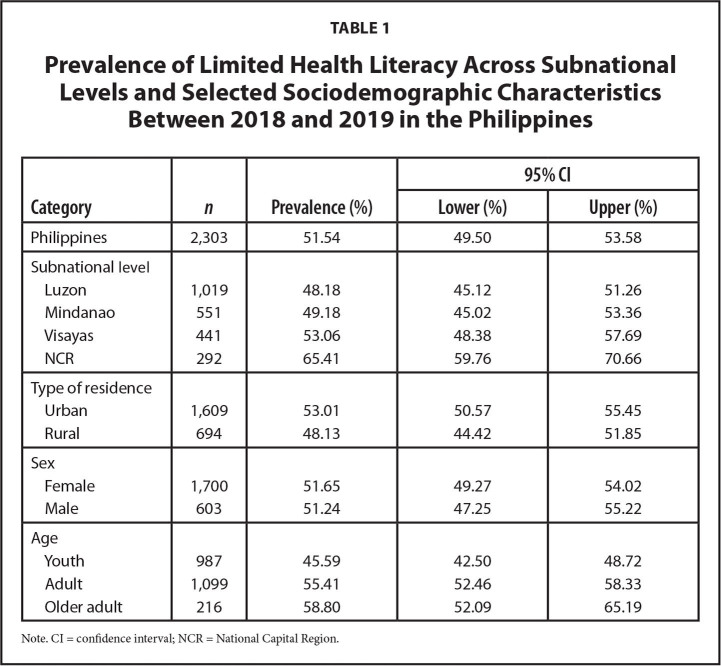
Prevalence of Limited Health Literacy Across Subnational Levels and Selected Sociodemographic Characteristics Between 2018 and 2019 in the Philippines

**Category**	***n***	**Prevalence (%)**	**95% CI**	

			**Lower (%)**	**Upper (%)**
Philippines	2,303	51.54	49.50	53.58

Subnational level				
Luzon	1,019	48.18	45.12	51.26
Mindanao	551	49.18	45.02	53.36
Visayas	441	53.06	48.38	57.69
NCR	292	65.41	59.76	70.66

Type of residence				
Urban	1,609	53.01	50.57	55.45
Rural	694	48.13	44.42	51.85

Sex				
Female	1,700	51.65	49.27	54.02
Male	603	51.24	47.25	55.22

Age				
Youth	987	45.59	42.50	48.72
Adult	1,099	55.41	52.46	58.33
Older adult	216	58.80	52.09	65.19

Note. CI = confidence interval; NCR = National Capital Region.

**Table C:**
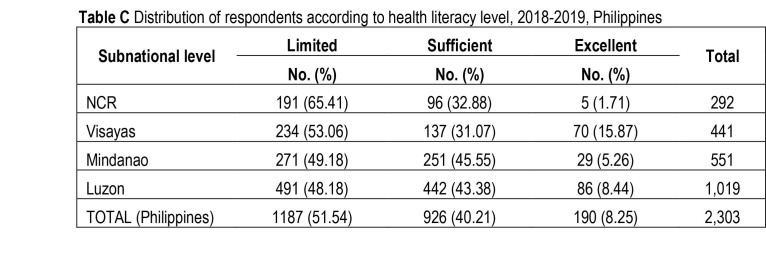
Distribution of respondents according to health literacy level, 2018–2019, Philippines

**Subnational level**	**Limited**	**Sufficient**	**Excellent**	**Total**
	**No. (%)**	**No. (%)**	**No. (%)**	
NCR	191 (65.41)	96 (32.88)	5 (1.71)	292
Visayas	234 (53.06)	137 (31.07)	70 (15.87)	441
Mindanao	271 (49.18)	251 (45.55)	29 (5.26)	551
Luzon	491 (48.18)	442 (43.38)	86 (8.44)	1,019
TOTAL (Philippines)	1187 (51.54)	926 (40.21)	190 (8.25)	2,303

**Figure 1.**
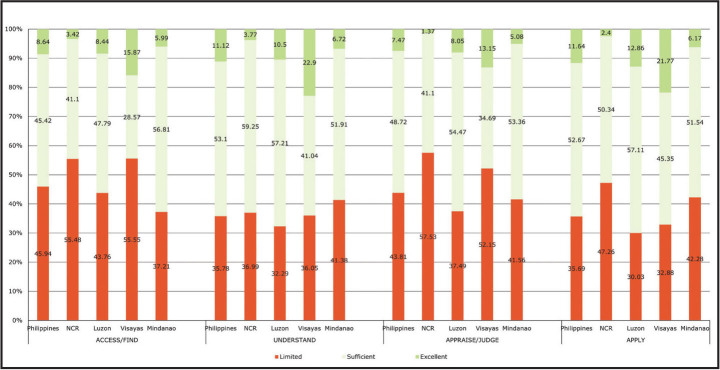
Distribution of respondents according to health literacy level by dimensions of health literacy and subnational levels between 2018 and 2019 in the Philippines.

As shown in **Table 2**, the nationwide prevalence of limited HL varied across the four dimensions, with the prevalence higher for accessing (45.9%) and appraising (43.8%), compared to understanding (35.8%) and applying (35.7%). This pattern was also true in Luzon and Visayas. In NCR, the dimension with the highest prevalence of limited HL was appraising health information, while in Mindanao it was applying health information (**Figure 1**).

**Table 2:**
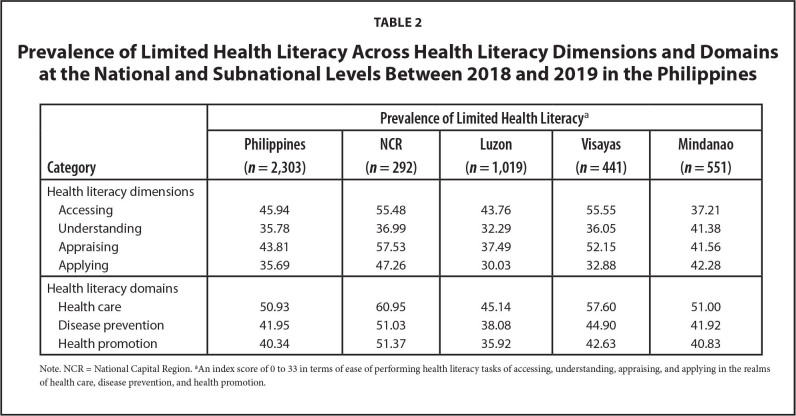
Prevalence of Limited Health Literacy Across Health Literacy Dimensions and Domains at the National and Subnational Levels Between 2018 and 2019 in the Philippines

**Category**	**Prevalence of Limited Health Literacy** ^a^				

	**Philippines (*n* = 2,303)**	**NCR (*n* = 292)**	**Luzon (*n* = 1,019)**	**Visayas (*n* = 441)**	**Mindanao (*n* = 551)**
Health literacy dimensions					
Accessing	45.94	55.48	43.76	55.55	37.21
Understanding	35.78	36.99	32.29	36.05	41.38
Appraising	43.81	57.53	37.49	52.15	41.56
Applying	35.69	47.26	30.03	32.88	42.28

Health literacy domains					
Health care	50.93	60.95	45.14	57.60	51.00
Disease prevention	41.95	51.03	38.08	44.90	41.92
Health promotion	40.34	51.37	35.92	42.63	40.83

Note. NCR = National Capital Region.

a An index score of 0 to 33 in terms of ease of performing health literacy tasks of accessing, understanding, appraising, and applying in the realms of health care, disease prevention, and health promotion.

**Figure 2.**
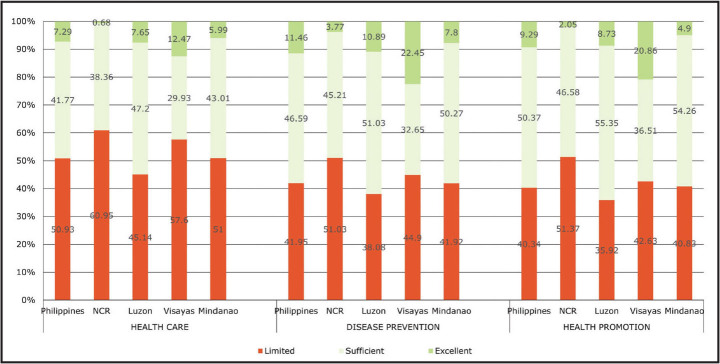
Distribution of respondents according to health literacy level by domains of health literacy and subnational levels between 2018 and 2019 in the Philippines.

The nationwide prevalence of limited HL differed across domains, with the health care domain having the highest prevalence at 50.9% (**Table 2**). The finding is consistent across the subnational levels. It is noteworthy that NCR has the highest prevalence of limited HL in all three domains (**Figure 2**).

The prevalence of limited HL varied across sociodemographic characteristics. The following variables did not show great absolute differences (≥10%) in limited HL to be considered of public health significance in terms of targeted interventions (**Table 3**): sex, civil status, and place of residence. The proportion of limited HL increased with age, whereas it decreased with increasing educational attainment. Moreover, respondents without health insurance had the highest proportion of limited HL. In addition, those without a relative with a medical background had a higher proportion of limited HL than those with relative(s) with medical background.

**Table 3:**
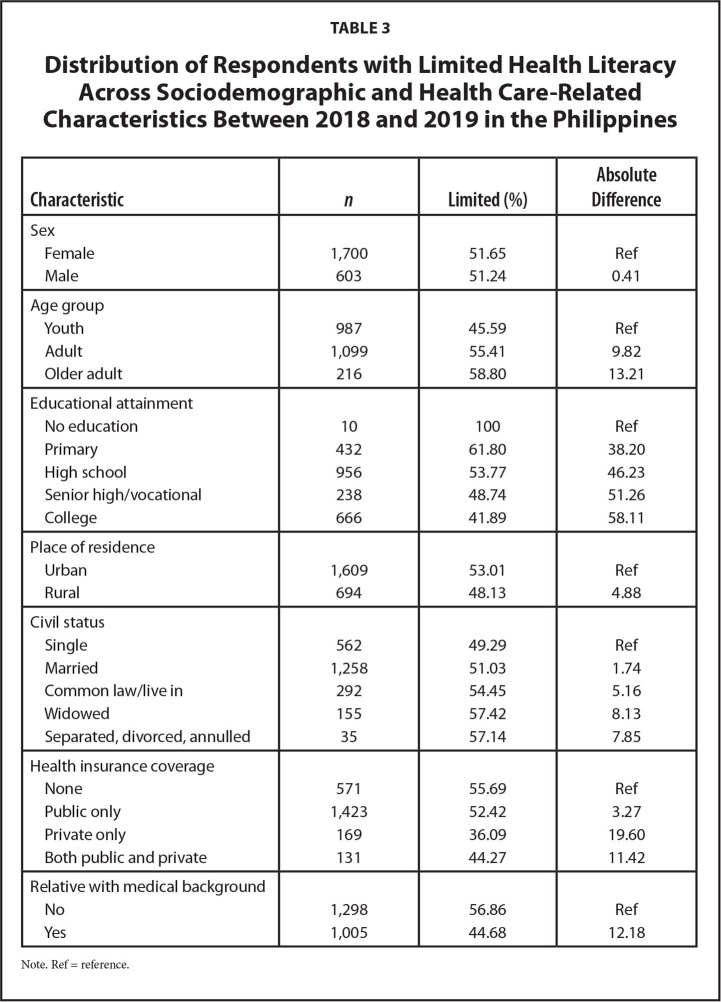
Distribution of Respondents with Limited Health Literacy Across Sociodemographic and Health Care-Related Characteristics Between 2018 and 2019 in the Philippines

**Characteristic**	***n***	**Limited (%)**	**Absolute** **Difference**
Sex			
Female	1,700	51.65	Ref
Male	603	51.24	0.41

Age group			
Youth	987	45.59	Ref
Adult	1,099	55.41	9.82
Older adult	216	58.80	13.21

Educational attainment			
No education	10	100	Ref
Primary	432	61.80	38.20
High school	956	53.77	46.23
Senior high/vocational	238	48.74	51.26
College	666	41.89	58.11

Place of residence			
Urban	1,609	53.01	Ref
Rural	694	48.13	4.88

Civil status			
Single	562	49.29	Ref
Married	1,258	51.03	1.74
Common law/live in	292	54.45	5.16
Widowed	155	57.42	8.13
Separated, divorced, annulled	35	57.14	7.85

Health insurance coverage			
None	571	55.69	Ref
Public only	1,423	52.42	3.27
Private only	169	36.09	19.60
Both public and private	131	44.27	11.42

Relative with medical background			
No	1,298	56.86	Ref
Yes	1,005	44.68	12.18

Note. Ref = reference.

## Discussion

About one-half (51.5%) of the study participants had limited HL, with the *access* dimension and the *health care* domain having the highest prevalence of limited HL; variations in HL levels were observed across sub-national levels.

In the Philippines, 19.7% of Filipino people age 5 years and older have a college education, and the basic literacy level is high (96.5%) (Philippine Statistics Authority, 2019). Despite this, the study found a high prevalence of limited HL. While literacy is an important factor in HL, it does not guarantee a high level of HL (Nutbeam, 2000). The Health Literacy Universal Precautions Handbook was conceptualized because it is difficult to tell one's HL level based on educational attainment; thus, health systems “should assume that all patients and caregivers may have difficulty comprehending health information and should communicate in ways that anyone can understand” (Brega et al., 2015, p. 1).

High prevalence of limited HL can be attributed to various factors, including low competencies of the population for engaging with health information, high expectations of the health system, or a combination of both (European Health Literacy Project Consortium, 2014; Nakayama et al., 2015). A community-based survey revealed that only 5.7% of the residents in an urban community in the Philippines had access to a Department of Health Cholera leaflet; understanding of the eleven concepts in the Cholera leaflet was also variable (Abis et al., 2015). Likewise, the high demands of the health system are also apparent in the Philippines. The Philippine Health System Review 2018 reported that health care system access is impeded by several factors: (1) limited number of practitioners and facilities, as well as poor geographic distribution of doctors and nurses; (2) high out-of-pocket cost for patients; and (3) barriers to health service access (Dayrit et al., 2018).

The burden of limited HL varied across subnational levels in the Philippines. Compared to the national level, the prevalence of limited HL in NCR (65.4%) was substantially higher, whereas the estimates in Luzon (48.2%) and Mindanao (49.2%) were lower. This implies differences in health promotion activities and their effectiveness and in health system demands (European Health Literacy Project Consortium, 2014; Nakayama et al., 2015; Nutbeam, 2017). There are reported variations in the quality of health services in different local government units in the Philippines at least partly due to the devolved health system (Dayrit et al., 2018; Solon and Herrin, 2017). The Department of Health has recognized the need to train health professionals on health promotion via field training facilities, to ensure the standard delivery of health promotion services (Department of Health, 2018).

Health information access had the highest prevalence of limited HL (45.9%) (**Table 2**). This is noteworthy considering that the process of engaging with sources of health information begins with accessing health information. This will trigger the rest of the steps, namely, understanding, appraising, and then applying the health information. As pointed out by Sørensen et al. (2012), this process generates the knowledge, skill, and motivation needed for an individual to navigate the health care system. Factors contributing to difficulties in access include the inadequate and poorly distributed health care professionals across and within regions, low utilization of health services, and a “mixed-health” system with increasing private health care services, without an effective regulatory mechanism for private for-profit health services (Dayrit et al., 2018). In this study, we found that in the last 12 months, 67.57% had not visited a health facility **(Table B)**, although these facilities are a major source of health information derived from printed health materials (Abis et al., 2015) and possibly also from provider-client interaction and televised health information. Lack of interaction with primary care physicians was also a cited reason for problems accessing health information in Japan (Nakayama et al., 2015).

Among the three domains, the highest prevalence of limited HL was in health care (50.9%). This implies that engaging with information about health care is more difficult than is the case with disease prevention or health promotion. Moreover, verbal health information from health providers on health care may be less understood than that of other domains. The reasons may include limited time available for health provider-patient interaction or communication skills of the health provider. This is in contrast with population HL levels, where the domain with the highest proportion of limited HL was disease prevention for Japan (Nakayama et al., 2015) and health promotion for other countries (Espanha & Ávila, 2016; Sørensen et al., 2012). It has been posited that personal experiences in the health care setting may enhance the HL skills of patients (Rolová et al., 2018). In this study, 67.6% of the respondents did not avail themselves of services at any health facility in the last 12 months, and 17.1% of the respondents had never consulted a health professional since age 13 years **(Table B)**. This may have contributed to the higher proportion of limited HL in the health care domain precisely because the lack of experiences as a patient may result in low knowledge on medical information and unfamiliarity with how to navigate the health care system.

The variables that showed absolute differences less than 10% (i.e., sex, civil status, place of residence) were inconsistently described in previous studies in terms of HL level across their respective categories. Some studies reported no significant difference, while in others, one category is higher than the other(s) (Haghdoost et al., 2019; Kayupova et al. 2017; Mahmoodi et al., 2019; Rasu et al., 2015; Schaeffer et al., 2017; Tiller et al., 2015; van der Heide, 2013).

Consistent with previous studies in other countries, there were noticeable differences in HL between age groups in this study (Abacigil et al., 2019; Schaeffer et al., 2017). The elderly showed the highest proportion of limited HL, which may be explained by physical impairment and cognitive decline related to advancing age (Chesser et al., 2016; Duong et al., 2015). Vision changes and hearing loss may impede information processing, while decreased motor function may inhibit adoption of necessary health behaviors. The elderly may also experience trouble in higher-order thinking skills, such as comprehension, comparison and contrast, and reasoning (Speros, 2009). In addition, age-cohort differences in health education during formal schooling contribute to disparities between age groups (Ashida et al., 2011; Xie et al., 2019). It is worth mentioning that the actual proportion of elderly individuals with limited HL may even be higher, because older adults with cognitive impairment were purposely excluded from the study.

The proportion of limited HL increased with decreasing level of educational attainment, with 100% having limited HL among those who have not entered school. Similar to prior studies (Duong et al., 2017; Jovanić et al., 2018; Tiller et al., 2015), these findings reflected the influence of formal education on HL by imparting health-related knowledge and forming skills essential for engaging with sources of information (Murray et al., 2008).

The prevalence of limited HL was higher among those without insurance coverage, which is consistent with previous studies (Briones, 2017; Sentell, 2012). This may denote that the complexity of insurance information and enrolment procedures may hinder those with limited HL to obtain health insurance (Sentell, 2012). Additionally, those without insurance have less use of health services due to higher out-of-pocket medical expenses (Foutz et al., 2017). This lack of experience with the health care system may lead to limited engagement with health information and consequently limited HL.

Those with public insurance had a higher proportion of limited HL compared to those with private insurance (absolute difference: 16.33). Studies comparing the HL levels of those with public or private insurance are limited. In a 2003 national survey in the United States, most uninsured participants, Medicaid beneficiaries (60%), and Medicare beneficiaries (57%) had below basic or basic HL, whereas only about 37% of the privately insured had the same level of HL (U.S. Department of Health and Human Services, 2009). This implies that insurance coverage alone cannot guarantee a meaningfully high HL (Vernon et al., 2007). Difference in personal health situation may play a role in one's ability to engage with sources of health information. In addition, those who have private insurance might be more likely to avail themselves of services from private facilities where the volume of clients and availability of health service providers and services is not a problem, unlike in government facilities. The quality of provider-client interaction may also play a role in effective communication, which is an important aspect of HL.

Finally, respondents who did not have a relative with a medical background had higher proportions of limited HL than those who did have a relative with a medical background. This was supported by the study of Pan et al. (2010), which observed higher HL among respondents with a family member working as a health professional. A health professional in the extended family may readily share health-related knowledge and persistently remind one of healthy behaviors (Chen et al., 2019). The nuanced spillover of health expertise may consequently lead to higher HL in their family members.

## Study Strengths and Limitations

Measures to minimize systematic error were put into place, from the design of the questionnaire to fieldwork supervision to data processing. They included the following: (1) localization, pre-testing, and validation of the HL questionnaire; (2) training of fieldwork teams on the various survey protocols; (3) data collectors carrying a brochure during data collection that served as a handy reference for the various survey protocols; (4) spot-checking of interviews by supervisors; and (5) using the CAPI method, which eliminated possible encoding errors encountered with the usual paper-and-pen interview and incorporated GPS for monitoring of interviewers to deter fabrication of interviews.

The study has some limitations. First, although the adapted questionnaires underwent localization, including an iterative process of translation analysis (Hall et al., 2018), it is still possible that there were changes in meaning. Second, while the National Health Literacy Survey result was based on a national sample of individuals, the Muslim religion of the Philippines was under-represented due to the exclusion of an entire Muslim region because of the poor peace and order situation during the data collection period. This limits the generalizability of the results. Also, the distribution of religion, employment, sex, and education do not adequately reflect the nationwide distribution based on the 2015 nationwide census. However, the adjusted estimates, ranging from 50.2% to 52.9%, are only slightly different from the unadjusted estimate of 51.5% **(Table D)**. Third, the random selection of one respondent per household would have inevitably resulted in unequal probability of selection per respondent, due to variation in household sizes. This could in principle have been corrected through the application of sampling weights; however, incomplete data on the weights made it impossible to compute for weighted estimates.

**Table D:**
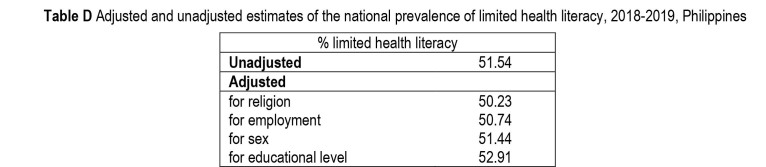
Adjusted and unadjusted estimates of the national prevalence of limited health literacy, 2018–2019, Philippines

% limited health literacy	
**Unadjusted**	51.54

**Adjusted**	

for religion	50.23
for employment	50.74
for sex	51.44
for educational level	52.91

## Conclusion

The majority of Filipino people nationwide have limited HL, and the prevalence estimates varied across HL dimensions, HL domains, subnational groupings, and sociodemographic characteristics. The results highlight the need for targeted interventions focusing on specific population subgroups with limited HL and on improvements in the information access dimension and in the health care domain of population HL. Further research can explore why some Filipino people perceive it to be difficult to perform various HL tasks and how the reported determinants of HL apply to the local setting.
